# Effect of the antirheumatic medication methotrexate (MTX) on biomechanical compressed human periodontal ligament fibroblasts (hPDLFs)

**DOI:** 10.1186/s12903-024-04092-1

**Published:** 2024-03-13

**Authors:** Claudia Welte-Jzyk, Vera Plümer, Sven Schumann, Andrea Pautz, Christina Erbe

**Affiliations:** 1 Department of Orthodontics, University Medical Center of the Johannes Gutenberg-University, 55131 Mainz, Germany; 2 Institute of Anatomy, University Medical Center of the Johannes Gutenberg-University Mainz, 55128 Mainz, Germany; 3 Department of Pharmacology, University Medical Center of the Johannes Gutenberg University, 55131 Mainz, Germany

**Keywords:** Human periodontal ligament fibroblast, Orthodontic compression, Rheumatoid arthritis, Methotrexate

## Abstract

**Background:**

The aim of this study was to investigate the in vitro effect of the antirheumatic drug methotrexate (MTX) on biomechanically compressed human periodontal ligament fibroblasts (hPDLFs), focusing on the expression of interleukin 6 (IL-6), as its upregulation is relevant to orthodontic tooth movement.

**Methods:**

Human PDLFs were subjected to pressure and simultaneously treated with MTX. Cell proliferation, viability and morphology were studied, as was the gene and protein expression of IL-6.

**Results:**

Compared with that in untreated fibroblasts, IL-6 mRNA expression in mechanically compressed ligament fibroblasts was increased (two to sixfold; ****p < 0.0001). Under compression, hPDLFs exhibited a significantly more expanded shape with an increase of cell extensions. MTX with and without pressure did not affect IL-6 mRNA expression or the morphology of hPDLFs.

**Conclusion:**

MTX has no effect on IL-6 expression in compressed ligament fibroblasts.

**Supplementary Information:**

The online version contains supplementary material available at 10.1186/s12903-024-04092-1.

## Background

In the course of orthodontic treatments, teeth migrate through the connective tissue surrounding them. Due to the application of an orthodontic force to a tooth, mechanical stress alters the structural properties of the periodontal ligament (PDL) between the tooth and the alveolar bone at cellular, molecular, and genetic levels leading to bone resorption at the pressure zone, while new bone is formed at the tension side [1–3]. This compression-tension theory is well accepted [4] as well as that remodelling of the extracellular matrix is essential to produce orthodontic tooth movement [5].

The mechanical load leads to an increase in various inflammatory mediators in the periodontal space. Inflammatory processes in the periodontium in response to orthodontic forces are prerequisites for remodelling activities and tooth displacement [6]. The PDL harbours a heterogeneous cell composition responsible for the perception of the mechanical load. The predominant cell type in the PDL is the human periodontal ligament fibroblast (hPDLF) surrounded by cementoblasts, osteoblasts, and osteoclasts [7]. In order to determine their relevance to orthodontic tooth movement, a simple and efficient model exists which describes the molecular responses to mechanical stress [8]. In this model, pressure is simply applied to the cells by placing a glass disc on the cell layer. Many working groups have already used this method [9–14] and found that inflammatory mediators such as prostaglandins, interleukins and the tumor necrosis factor-α superfamily were increased in the PDL during orthodontic treatment [4, 6, 15, 16].

Juvenile idiopathic arthritis (JIA) is the most common rheumatic disease among children and adolescents, with an average prevalence of 1 in 1000 children aged 0–15 years across Europe [17]. It is an autoimmune inflammatory disease with synovial immune cell infiltration characterized by joint stiffness, swelling and pain [18]. All joints, including the temporomandibular joint (TMJ), may be affected [17, 19]. The shape and structure of the TMJ are compromised, so these patients often have a skeletal class II malocclusion and an anterior open bite [20]. Both, an anti-inflammatory as well as orthodontic treatment are necessary to successfully relieve symptoms of JIA, control the disease, and improve abnormalities of the dental apparatus to prevent temporomandibular joint replacement with an endoprosthesis.

Methotrexate (MTX) is most frequently used for reducing destruction to the TMJ and therefore minimising associated deformities, especially in patients with polyarticular JIA [20, 21]. MTX is a folic acid antagonist that competitively and reversibly inhibits dihydrofolate reductase, which is required for the formation of purines and thymidines. In addition, MTX also inhibits the thymidilate synthase, which catalyzes pyrimidine synthesis. By this mode of action, MTX inhibits de novo synthesis of DNA and RNA and therefore the proliferation of cells [22, 23]. At low doses, MTX has an anti-inflammatory effect and is used as a disease-modifying antirheumatic drug (DMARD) in the treatment of JIA and other inflammatory autoimmune diseases [24]. MTX modifies autoimmunological inflammatory processes during JIA via multiple mechanisms, including adenosine mediated anti-inflammatory effects, increased apoptosis of T cells and the reduction of cell proliferation [25–27]. Despite the anti-inflammatory effect of MTX treatment, MTX did not improve the periodontal condition of RA patients [28] although marginal differences in the prevalence of select potentially pathogenic bacteria were detected [25]. MTX treatment in a mouse model of antigen-induced arthritis led to greater dominance of the health-associated bacteria as well as significantly less periodontal bone loss [29]. Fibroblasts are known to be the major cellular source of inflammatory cytokines in inflammatory diseases such as rheumatoid arthritis. Through their production of IL-6, these cells contribute significantly to the regulation of inflammation [30, 31]. The release of pro-inflammatory cytokines from periodontal fibroblasts was increased after MTX treatment without microbial stimulus, but was reduced after MTX treatment after stimulation by *F. nucleatum* [32]. Human gingival fibroblasts (HGF) exposed to different concentrations of MTX showed evaluated interleukin (IL)-6 production [33]. To our knowledge, the effect of MTX on hPDLFs under pressure is still unclear.

The aim of our study was to investigate the impact of MTX treatment on static compressed hPDLFs in vitro. We focussed on morphological changes as well as changes in the expression of IL-6 due to its relevance in orthodontics.

## Methods

### Cell culture

Commercially acquired human periodontal ligament fibroblasts (HPDLFs, Lonza, Basel, Switzerland) were grown in Dulbecco’s modified Eagle medium (DMEM; Thermo Fisher Scientific, Carlsbad, CA, USA) supplemented with 4.5 g/L glucose, 10% fetal bovine serum (Thermo Fisher Scientific, Carlsbad, CA, USA), 100 U/ml penicillin, 100 µg/ml streptomycin and 50 mg/L L-ascorbic acid (Sigma Aldrich, Munich, Germany) at 37 °C, 5% CO_2_, and 95% humidity. For all the experiments, passages four to eight were used for the experimental setups.

### Mechanical compression

HPDLFs were stimulated by compressive force as previously described [8]. Briefly, glass discs (2 g/cm^2^, Glas Schwarz, Mainz, Germany) were placed in 6-well plates for different durations (4, 12, 24 and up to 72 h) at 37 °C, 5% CO_2_, and 95% humidity. In 12-well plates, the application of compressive force was performed by using circular coverslips (diameter of 20 mm, NeoLab, Germany) loaded with metal discs of 20 mm in diameter containing a central hole to visualise the cells by life cell imaging.

### MTT cell vitality assay

The viability of the hPDLF was detected directly after applying compressive force with a colorimetric assay (3-(4.5-dimethyl-2-thiazolyl)-2.5-diphenyl 2H-tetrazolium bromide (MTT), Sigma Aldrich, Munich, Germany) according to the manufacturer’s protocol. Viable cells metabolize tetrazolium bromide into formazan, which was measured photometrically at a wavelength of 550 nm (VersaMax Microplate Reader; Molecular Devices, Sunnyvale, USA). Each experiment was performed with three replicates and each approach was analysed in triplicate. The viability of the control cells was set to 100%.

### Proliferation

Cell proliferation was assessed based on the increase in confluence observed with a life cell imaging device (IncuCyte, Sartorius).

### Scanning electron microscopy (SEM)

Cells were seeded in six well plates on glas slides for electron microscopy (tissue culture coverslips, diameter 13 mm, Sarstedt). After 24 h, enough time had elapsed for the cells to become adherent, the cells were treated with medication and/or compression force for 24 h. Thereafter, the cells were fixed with 4% PFA and 2.5% glutaraldehyde. After the cells were washed with PBS, a secondary fixation with osmium tetraoxide (1%, Carl Roth, Deutschland) was applied for 60 min at room temperature. The cells were washed and dehydrated with a graded ethanol series (25%, 50%, 75%, 95%, 100%), followed by critical-point drying (CPD). Electron micrographs were taken on cells sputtered with gold in an argon atmosphere (Leica EM ACE 200).

### Ribonucleic acid (RNA) extraction and quantitative polymerase chain reaction (PCR)

Messenger RNA (mRNA) was extracted using the commercially available RNeasy® Mini Kit (Qiagen GmbH, Hilden, Germany), according to the manufacturer’s protocol, which included a DNAse digestion step to remove the genomic DNA from the mRNA samples. The quantity and quality of the isolated mRNA were evaluated using a NanoDrop Spectrophotometer (pegLab Biotechnologie GmbH, Erlangen, Germany) and equal amounts of mRNA was converted to complementary DNA (cDNA) via the iscript cDNA Synthesis Kit according to the manufacturer’s protocol (Bio-Rad Laboratories, Hercules, USA). PCR primers for the quantitative detection of the cDNA levels were constructed with the NCBI nucleotide library and Primer 3-design (Table 1). All primers had been matched to the mRNA sequences of the target genes. As an appropriate housekeeping gene for normalizing the data via reverse transcription quantitative PCR (RT-qPCR) we used the ribosomal protein L22 (RPL22) gene which was found to be the most suitable for compressed ligament fibroblasts [11, 34, 35]. For reverse transcriptase polymerase chain reaction (RT-PCR) amplification, a reaction mixture was made containing SYBR Green Supermix (Bio-Rad Laboratories, Hercules, USA), paired primers, and a defined amount of template cDNA. Quantitative RT-PCR was performed with a thermal cycler (CFX Duet RT-PCR Detection System; Bio-Rad Laboratories, Hercules, USA) and CFX Maestro software version 2.3 (Bio-Rad Laboratories, Hercules, USA). The initial denaturation was induced at 95 °C for 5 min, followed by 40 cycles at 95 °C for 30 s (denaturation), 60 °C for 30 s (annealing) and 72 °C for 20 s (elongation). For each specific primer and real-time PCR, the efficiency was calculated based on the SYBR Green fluorescence curves and the standard dilution series by CFX Maestro software version 2.3 (Bio-Rad Laboratories, Hercules, USA). For gene expression analysis, the ΔΔCtq method was used [36]. According to this method the C(T) values of IL-6 and HIF-1α mRNA in each sample were normalized to the C(T) values of RPL22 mRNA in the same sample.

**Table 1:** Primer sequences for the target and reference genes (RPL22)

Gene		5´-Forward primer-3´	5´-Reverse primer-3´
HIF 1α	Hypoxy inducible factor 1α	GCAGCTACTACATCATCTTCTT	CAGCAGTCTACATGCTAAATCA
IL-6	Interleukin 6	TGGCAGAAAACAACCTGAACC	CCTCAAACTCCAAAAGACCAGTG
RPL22	Ribosomal protein L22	TGATTGCACCCACCCTGTAG	GGTTCCCAGCTTTTCCGTTC

### Enzyme-linked immunosorbent assay (ELISA)

The protein production of IL-6 was quantified by using a commercially available kit (R&D Systems, Inc., Minneapolis, USA). The culture supernatants were collected after treatment and analysed in accordance with the manufacturer’s instructions. The optical density was determined using a microplate reader (VersaMax Microplate Reader; Molecular Devices, Sunnyvale, USA) set to 450 nm, and the wavelength correction was set to 540 nm. Each assay was performed in triplicate. Supernatants of untreated cells were used as controls.

### Statistical analysis

To detect any difference between the groups, an unpaired t-test or one-way ANOVA with the post hoc Tukey test was used. A p value of < 0.05 was considered to indicate statistical significance. cDNA from individual cell experiments was analysed via triplicate PCR. The relative expression levels of each mRNA were evaluated by using a modification of the ΔΔCT method [36]. The standard error of the mean was calculated using GraphPad Prism (Dotmatics; Boston, USA).

## Results

### Cell proliferation and viability

**Fig. 1:**
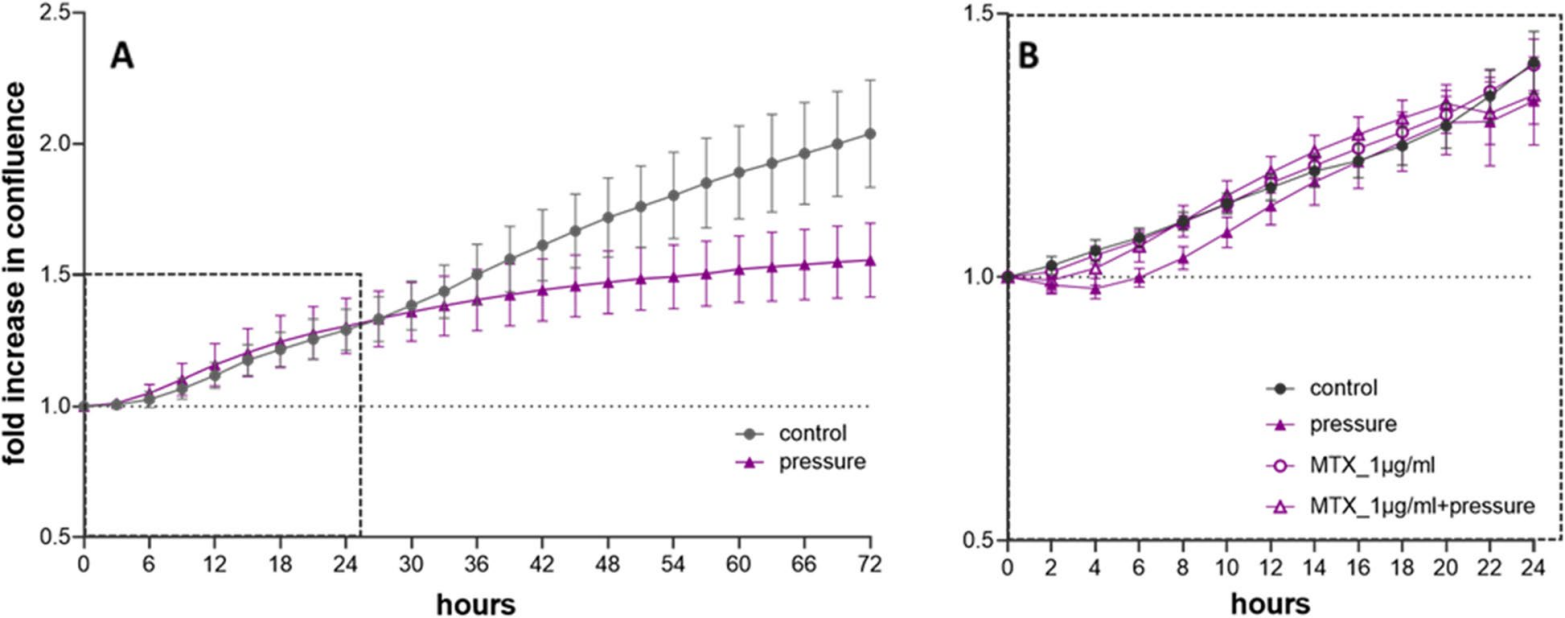
Proliferation of hPDLFs under pressure. **A** Increase in confluence over time—control versus pressure (*n* = 6) **B** Increase in confluence of cells treated with 1 µg/ml MTX and pressure compared to control cells (*n* = 4). Live cell imaging revealed that the viability of untreated and MTX treated cells was comparable with and without pressure (Additional file 1). MTX treatment had no influence on cell viability

The effect of biomechanical stress on cell proliferation was monitored by live cell imaging as an increase in confluence. Cells in both experimental setups, the unloaded control group and the biomechanical treatment group were proliferating (Fig. 1A). The proliferation of cells under pressure was almost comparable to that of the controls after up to 24 h. After 24 h, however, the growth rate of the cells under pressure was lower than that of the control cells (Fig. 1A). Consequently, all further experiments were performed at a maximum time interval of 24 h. Treatment of cells with 1 µg/ml MTX did not impair the proliferation rate (Fig. 1B).

### Cell morphology

**Fig. 2:**
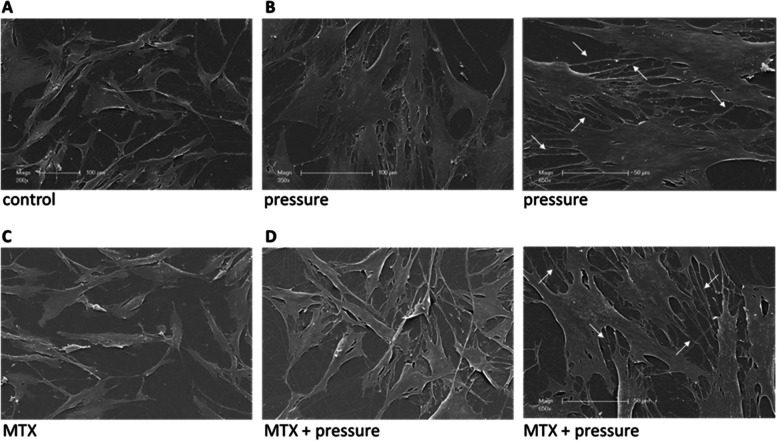
Morphology of hPDLFs under pressure. After 24 h of pressure, the fibroblasts appeared flattened and expanded and showed many extensions (stolons) in the electron micrographs. **A** Control hPDLFs (bar = 100 µm), **B** HPDLFs under pressure (bar = 100 µm, 50 µm, stolons marked by white arrows), **C** HPDLFs treated with MTX (1 µg/ml) (bar = 100 µm),** D** HPDLFs treated with MTX (1 µg/ml) and pressure (bar = 100 µm, 50 µm, stolons marked by white arrows)

Compared with control cells, the compressed hPDLFs were clearly flatter and more extended (Fig. 2A versus Fig. 2B). In addition, pressurized cells exhibited significantly more and longer extensions that appeared to connect the individual cells (Fig. 2B, indicated with white arrows). MTX had no effect on cell morphology (Fig. 2A versus 2C, 2B versus 2D).

### mRNA expression

**Fig. 3:**
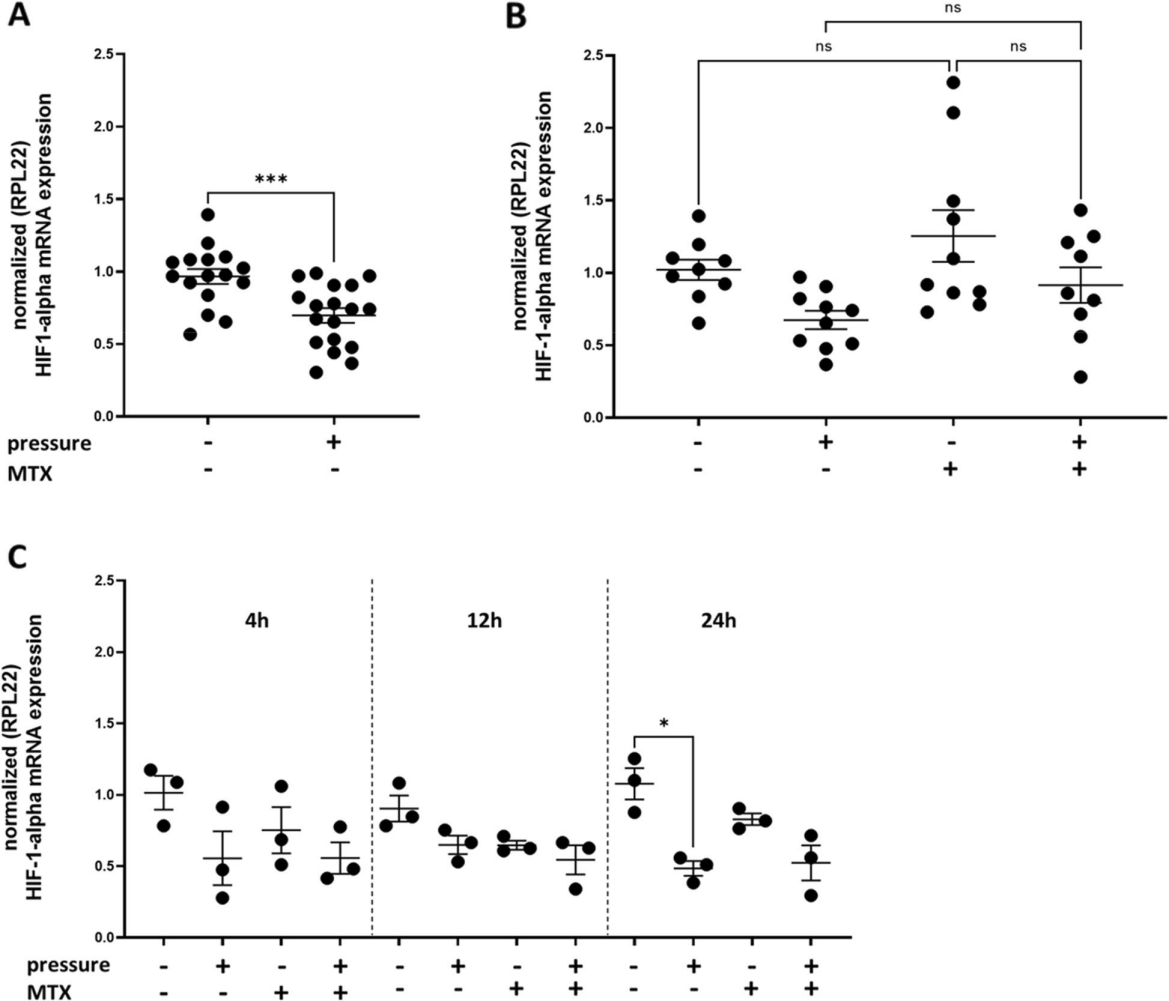
Hypoxia inducible factor (HIF)-1α mRNA expression under pressure and MTX treatment. **A** HIF-1α mRNA expression was reduced under pressure (*n* ≥ 15, *** p < 0.001, unpaired t-test) **B** HIF-1α mRNA expression was not influenced by MTX treatment (*n* > 9, ns, one-way ANOVA) **C** Mechanical stress reduced HIF-1α mRNA expression over time since there was no reduction at 4 or 12 h, this change occurred only at 24 h (*n* = 3, * p < 0.05, one-way ANOVA). The values are presented as the mean ± SEM. For gene expression analysis, the ΔΔCtq method was used [36]. As a housekeeping gene, RPL22 was used because it works quite well for compression studies [11, 37]

To exclude the possibility of cells being deprived of oxygen after 24 h, the mRNA expression of hypoxia inducible factor (HIF)-1α was examined by quantitative RT-PCR (qRT-PCR). HIF-1α was not increased in cells which were under pressure for 24 h, on the contrary, the mechanical stress caused a significant reduction in HIF-1α mRNA expression (***p < 0.001, unpaired t-test; Fig. 3A). No differences were detected between control and fibroblasts treated with MTX (1 µg/ml), nor between fibroblasts treated with MTX with or without additional mechanical stress (ns, one-way ANOVA, Fig. 3B). Mechanical stress reduces HIF-1α mRNA expression over time since there was no reduction at 4 and 12 h, this only occurs at 24 h (*n* = 3, * p < 0.05, one-way ANOVA, Fig. 3C).

**Fig. 4:**
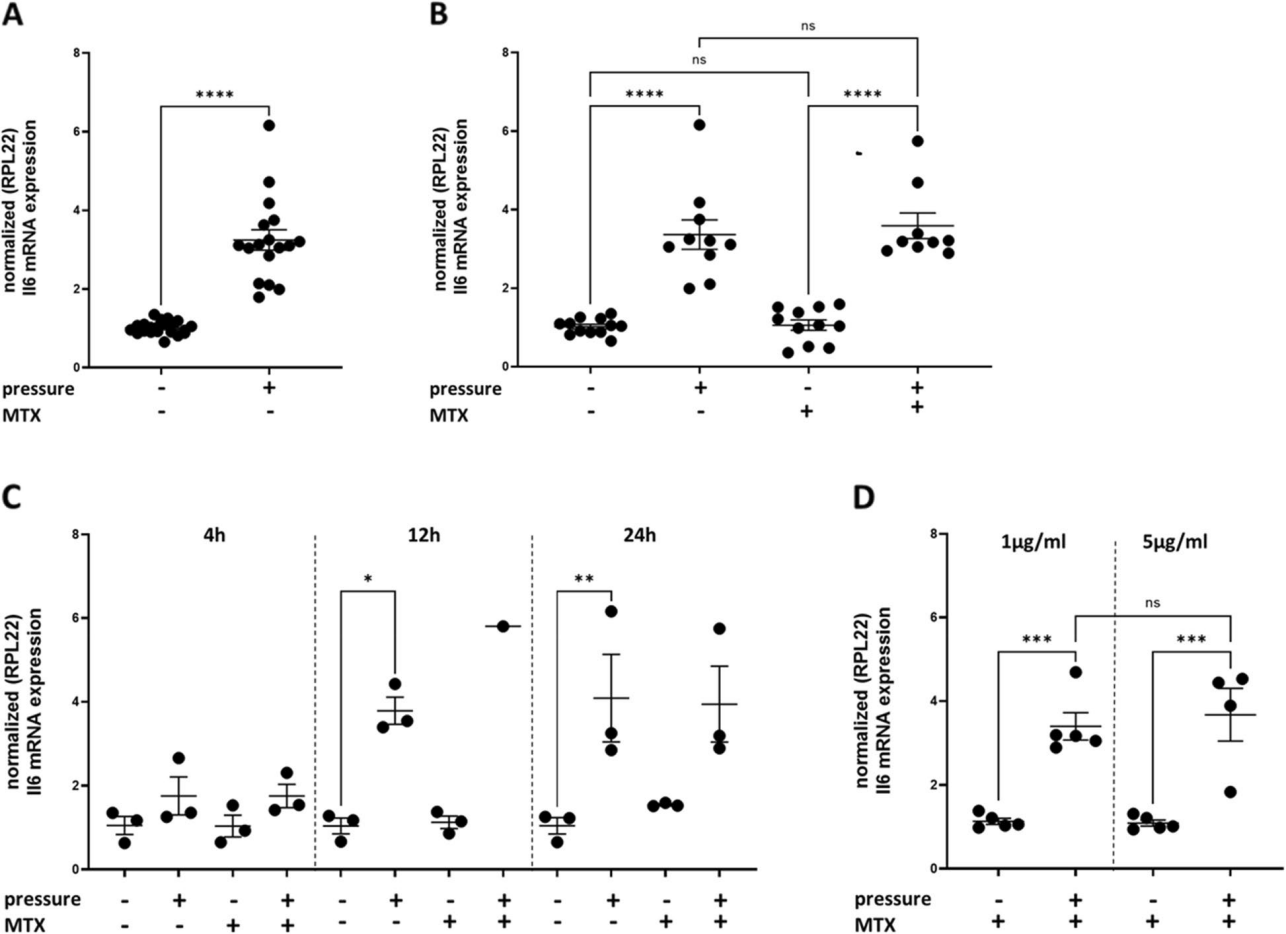
Interleukin 6 (IL-6) mRNA expression under pressure and MTX treatment. **A** IL-6 mRNA expression was increased under pressure (*n* ≥ 15, **** p < 0.0001, unpaired t-test) **B** IL-6 mRNA expression was not influenced by MTX treatment (1 µg/ml) (*n* ≥ 12, ns, one-way ANOVA). IL-6 mRNA expression was increased in MTX-treated fibroblasts under pressure to the same extent as in fibroblasts under mechanical compression without MTX (*n* ≥ 9, **** p < 0.0001, one-way ANOVA) **C** Mechanical stress increased IL-6 mRNA expression over time in mechanically compressed fibroblasts without and with MTX treatment (*n* = 3, * p < 0.05-** p < 0.01, one-way ANOVA). **D** IL-6 mRNA expression did not increase further inr mechanically compressed fibroblasts treated with higher concentrations of MTX (5 µg/ml). The values are presented as the mean ± SEM. For gene expression analysis, the ΔΔCtq method was used [36]. As a housekeeping gene, RPL22 was used because it works quite well for compression studies [11, 37]

IL-6 mRNA expression increased under pressure (*n* > 15, **** p < 0.0001, unpaired t-test, Fig. 4A). IL-6 mRNA expression was not influenced by MTX treatment (1 µg/ml) MTX (*n* > 9, ns, one-way ANOVA, Fig. 4B). IL-6 mRNA expression was increased in MTX-treated fibroblasts under pressure to the same extent as in fibroblasts under mechanical compression without MTX (*n* > 9, **** p < 0.0001, one-way ANOVA, Fig. 4B). Mechanical stress increases IL-6 mRNA expression over time in compressed fibroblasts without and with MTX treatment (*n* = 3, * p < 0.05-** p < 0.01, one-way ANOVA, Fig. 4C). IL-6 mRNA expression did not increase further in mechanically compressed fibroblasts treated with higher concentrations of MTX (5 µg/ml) (Fig. 4D).

### IL-6 protein expression

**Fig. 5:**
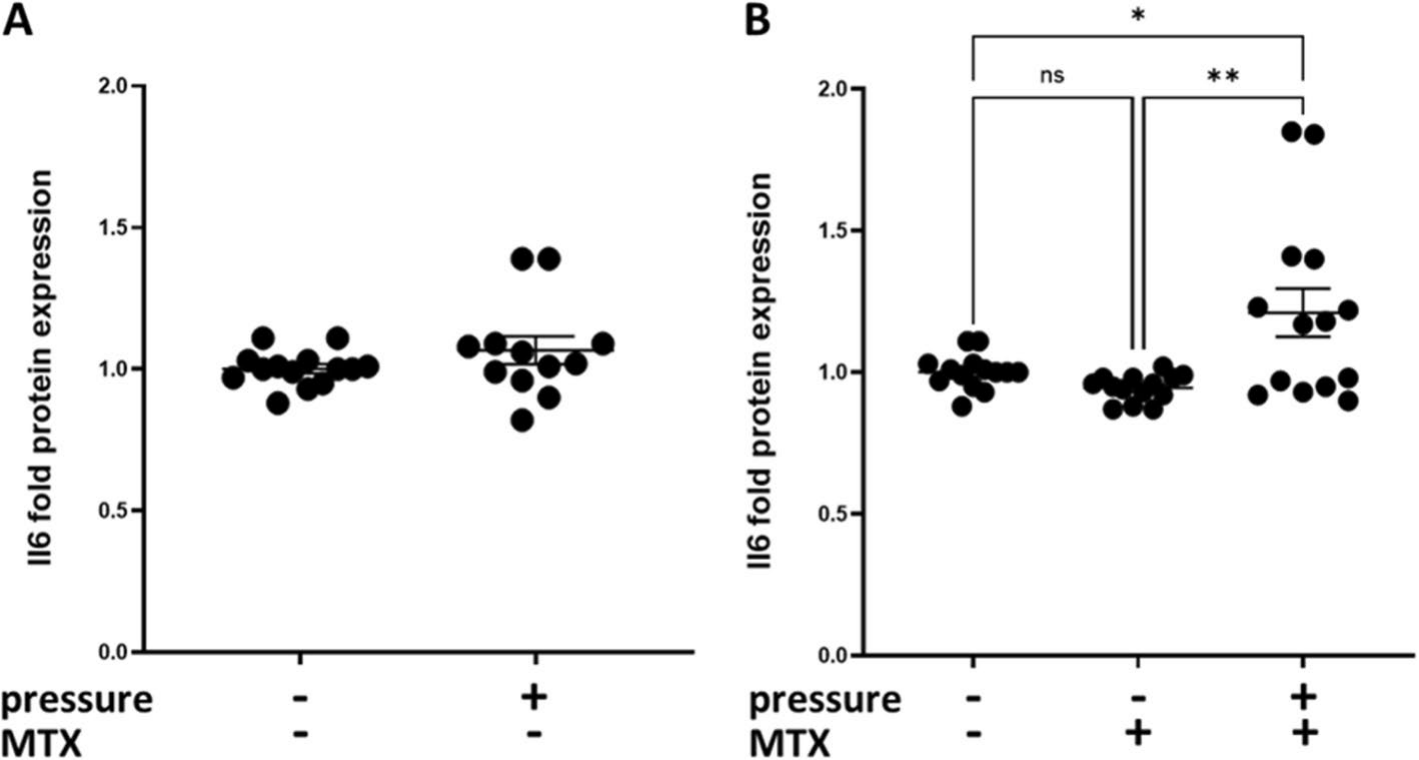
Interleukin 6 (IL-6) protein release under pressure and MTX treatment. **A** IL-6 protein release did not significantly differ under pressure (*n* ≥ 12, ns, unpaired t-test) **B** IL-6 protein release was not influenced by MTX treatment (1 µg/ml) (*n* ≥ 12, ns, one-way ANOVA), but was slightly increased under concurrent pressure application (*n* ≥ 12, ** p < 0.001, one-way ANOVA). The values are presented as the mean ± SEM

IL-6 protein release did not significantly different under pressure (*n* ≥ 12, ns, unpaired t-test, Fig. 5A) and was not influenced by MTX treatment (1 µg/ml) (*n* > 9, ns, one-way ANOVA, Fig. 5B), but was slightly increased under concurrent pressure application and MTX treatment (*n* ≥ 12, ** p < 0.001, one-way ANOVA, Fig. 5B).

## Discussion

To our knowledge, this is the first study to investigate the effect of the antirheumatic drug MTX on ligament fibroblasts in-vitro with simultaneous biomechanical stress.

Many in vitro studies have investigated the mechanism by which mechanical signals are transduced into biological signals that regulate bone homeostasis via periodontal ligament fibroblasts during orthodontic treatment [38]. Different methods for applying mechanical forces to cells have been established, and the effects of these forces on the osteogenic and osteoclastogenic properties of ligament fibroblasts have been studied [39]. Kanzaki et al. 2002 [8], and adopted by many groups interested in orthodontics [9, 14, 40], developed a simple method to apply pressure to fibroblasts. The pressure on cells is achieved simply by placing a glass disc on the cell layer. A compressive force of 2 g/cm^2^ is sufficient and has been tested in several studies [8, 41]. Nevertheless, it must be considered that the force magnitude possible in the in vitro simulation corresponds only to a limited extent to the force conditions that occur during orthodontic treatment in the root region of moving teeth [42].

Using this method, we observed the cell proliferation of ligament fibroblasts by life cell imaging over a 72-h period and found that the cell proliferation rate to be almost comparable with and without pressure up to 24 h (Fig. 1A). After this 24-h interval, the proliferation was more pronounced in the control group not subjected to pressure. This was also found in other studies [15]. For this reason, all further experiments, including those involving MTX treatment, were performed for a maximum duration of 24 h and the proliferation rate was comparable for all the approaches (Fig. 1B, control and MTX treatment with and without pressure). Moreover, cell viability could be determined by cell observation over time and no difference in viability was detected among the differently treated cells within the 24 h used for the experiments (Additional file 1). However, the metabolism of the mechanically compressed fibroblasts seems to be slightly lower than that of control cells, as revealed by the MTT test. The results of MTT tests performed by other groups vary, for example, some studies have shown a reduced cell number and viability during compressive force application [43–45], while others have shown no significant difference compared to those of the control [46]. However, treatment with MTX had no effect on cell viability. We had the impression that in the process of lifting the glass plates off the cell layer prior to the MTT test, the cells detached from the cell layer and consequently were lost for the viability measurement. Therefore, we considered the MTT test to be unsuitable for determining the viability of mechanically compressed cells.

During the monitoring of cell vitality by live cell analysis, a change in the morphology of the cells under pressure was noticeable (Additional file 1). Electron micrographs further illustrate this phenomenon (Fig. 2). Fibroblasts under pressure exhibit an extended flatter appearance. It is also impressive that the cells under pressure form more and longer extensions to contact each other (Fig. 2B, D). Such an increase in the quantity of cell junctions to neighbouring cells was also found in other studies [37]. Cell junctions establish and maintain intercellular contact between cells and are involved in key cellular functions, such as barrier formation, proliferation, migration, survival, and differentiation [47].

The slight decrease in the metabolism of the compromised cells prompted us to verify whether the cells were adequately oxygenated during the 24-h experiments under the glass plates. For this purpose, we analysed the expression of the hypoxia inducible factor (HIF)-1α, as hypoxia results in the upregulation of HIF-1α [48], leading to a hypoxia-induced metabolic shift [18].

In our study, HIF-1α was not increased in ligament fibroblasts under pressure, on the contrary, the mechanical stress caused a significant reduction in HIF-1α mRNA expression (*p* < 0.001, unpaired t-test; Fig. 3A). This finding is in contrast to those of other studies [46]. No increase in HIF-1α mRNA expression was observed at earlier time points (ns, one-way ANOVA; Fig. 3C). Ligament fibroblasts do not appear to be deprived of oxygen, but how can the decrease in HIF-1α mRNA be explained? Several studies have shown HIF-1α mRNA to be destabilized early within 3 h [49]. Other studies discuss that the confluence plays a crucial role in regulating target genes in compression experiments. Thus, the mRNA expression of several inflammatory markers and bone remodelling markers significantly differ between 60 and 100% confluent cells [37]. Indeed, many markers (e.g. Il-1a, Rankl and Ocn) are regulated in an opposite directions at variable confluences, especially after 48 h of loading, which may reverse the outcome of an entire experiment [37].

Moreover, what does this mean for orthodontic treatment, which seems to require a hypoxic environment to a certain extent? [50, 51] HIF-1α is discussed to enhance the bone-resorbing activity of mature osteoclasts [52] and as other studies found, this process is known to precede osteoclastogenesis [43, 53]. For orthodontic tooth movement the mechanical deformation of ligament fibroblasts seems to be more important than hypoxia [43].

Since we observed a morphological change in the compressed cells (Fig. 2), we assumed that the fibroblasts were activated in a manner comparable to orthodontic treatment. Upregulation of inflammation-related cytokines is considered a prerequisite for successful orthodontic treatment [2, 54]. We therefore examined the expression of the inflammation-related cytokine IL-6 as it is found to be increased in ligament fibroblasts due to mechanical stress by several research groups [15, 13, 55, 56]. We also found that IL-6 mRNA expression was upregulated due to mechanical compression (Fig. 4). The induction of IL-6 mRNA upregulation increased over time (Fig. 4C). After 24 h, the gene expression was significantly higher in compressed ligament fibroblasts (3.25 ± 0.26 fold; **** p < 0.0001) (Fig. 4A). Similar increases in IL-6 mRNA expression were also found in compressed ligament fibroblasts in other studies [10], in which the highest levels were found to be maximal after 48 h of pressure application in most studies [13, 15, 37]. In addition, the confluence of the cell layer seems to play a crucial role in the regulation of IL-6 in loading compression experiments [37] and therefore strict compliance with cell confluence is essential for meaningful and comparable results. In our studies, we used 70.000 cells/well and compression was started after 24 h of adhesion at which point the cells reached approximately 70% confluence.

Whether IL-6 gene and protein expression is upregulated in mechanically compressed ligament fibroblasts compared with control cells according to the method of Kanzaki [8] varies widely in the literature [10, 15, 42, 46, 55–58]. In our opinion, the reason for this can be attributed to the method used. Depending on the viability of the cells at the beginning of the experiment, the cell passage and cell density, the cells responded differently to the application of the glass plates.

Based on our experience that the proliferation of ligament fibroblasts was reduced after 24 h of pressure application (Fig. 1A), and because we observed a significant increase in IL-6 mRNA expression already after 24 h, we kept this time point for all further experiments. However, this time span may have been too short to represent significant upregulation of protein expression (Fig. 5A).

Rheumatoid arthritis (RA) can be treated with drugs that influence inflammatory factors. The level of inflammatory factors such as IL-6 are reduced. What significance does this have for orthodontic treatment, where mechanical pressure leads to artificial inflammation, which is considered a prerequisite for the success of orthodontic treatment?

The guidelines for the treatment of rheumatoid arthritis recommend MTX, a folic acid antagonist and traditional disease-modifying anti-rheumatic drug (DMARDs) as agent for the first choice [59]. RA is a disease triggered by an overactive immune system. The level of inflammatory mediators such as IL-6 are elevated [30, 31]. Fibroblasts are the key cellular source of inflammatory cytokines and chemokines that enable chronic tissue inflammation. Treatment of RA with immunosuppressive therapies is scheduled to control inflammation. MTX has strong anti-inflammatory effects [60]. The effect of MTX on fibroblast-like synovial cells was confirmed by the suppression of IL-6 production [61]. Others found a minor effect on the IL-8 level [32].

A number of in vitro studies suggest that MTX inhibits the proliferation of fibroblasts and induces their apoptosis [60]. We found no impairment in the proliferation of the ligament fibroblasts after MTX treatment up to 5 µg/ml MTX as shown in other studies as well [32, 62]. In addition, MTX did not significantly affect IL-6 mRNA expression compared to that in untreated fibroblasts, regardless of whether the cells were compressed (Fig. 4B, C). The expression of other inflammatory cytokines (IL-8 and PGE2) as well as markers of orthodontic tooth movement (RANKL and OPG) during MTX treatment will be investigated in future studies.

### Limitations and consideration

In vitro studies represent snapshots in an artificial system, which often could not be transferred directly to the clinical situation. They are isolated insights into events, whereas in an organism many cells work together and influence each other. For example, the half-life of MTX is only up to 10 h [27, 63], whereas the effect of MTX on organisms is prolonged by intracellular polyglutamatization through folypolyglutamate synthetase. Patients are therefore given weekly doses of MTX [27]. Since the proliferation rate of the hPDLF under pressure had already decreased after 24 h in comparison to that of the control (Fig. 1A), we limited our experiments to this time interval. 24 h of pressure is sufficient to initiate the artificial inflammation required for orthodontic tooth movement [13, 15, 55, 56] and therefore we investigated the effect of MTX on the expression of the inflammatory IL-6, which is upregulated early in the response to pressure. Parallel MTX administration did not influence the release of the inflammatory mediator IL-6 within 24 h. Since it can take several weeks for MTX to show an effect in patients [63, 64], we do not want to draw any conclusions as to whether MTX treatment has an effect on orthodontic treatment efficacy. Proliferation of the cells over several weeks cannot be achieved in vitro. In an animal model, the administration of MTX significantly increased orthodontic tooth movement [62], while many NSAIDs reduced movement speed [63]. On the other hand, MTX can reduce microorganism-stimulated release of IL-8 and IL-1β by periodontal fibroblasts within 18 h [32]. In vivo, the effect of MTX on other cells, such as immune cells or oral microorganisms, are also of importance or more important than those on hPDLFs.

## Conclusions

The primary aim of this study was to verify the critical role of antirheumatic MTX treatment in hPDLFs under biomechanical stress. We found that hPDLFs under pressure exhibit a flatter appearance with an increase in the number of connections between cells. IL-6 gene expression was upregulated in mechanically compressed cells. MTX had no significant effect on morphology or IL-6 gene expression regardless of whether the cells were compressed or not. However, since it can take several weeks for MTX to show an effect in patients, we do not want to draw conclusions as to whether treatment with MTX has an effect on orthodontic treatment. For this purpose, additional clinical studies are essential.

### Supplementary Information

**Additional file 1.** The viability of hPDFLs seems not to be influenced by 24 h of pressure or/and MTX treatment. Cell visualization over 24 h: sequence of 13 images for each treatment – control, pressure, MTX and MTX + pressure.

## Data Availability

The datasets used and/or analysed during the current study are available from the corresponding author on reasonable request.

## Abbreviations

ANOVA: Analysis of variance
CPD: Critical-point drying
DMARD: Disease-modifying anti-rheumatic drug
DMEM: Dulbecco’s modified Eagle medium
DNA: Desoxyribonucleic acid
ELISA: Enzyme-linked immunosorbent assay
HIF: Hypoxy inducible factor
JIA: Juvenile Idiopathic Arthritis
hPDLF: Human periodontal ligament fibroblast
IL: Interleukin
MTT: (3-(4.5-Dimethyl-2-thiazolyl)-2.5-diphenyl 2H-tetrazolium bromide
MTX: Methotrexate
OCN: Osteocalcin
PCR: Polymerase chain reaction
PDL: Periodontal ligament
PFA: Paraformaldehyde
RA: Rheumatoid arthritis
RANKL: Receptor activator of nuclear factor kappa-Β ligand
RPL22: Ribosomal protein L22
RNA: Ribonucleic acid
TMJ: Temporomandibular joint
